# Suppression of Stochastic Domain Wall Pinning Through Control of Gilbert Damping

**DOI:** 10.1038/s41598-017-17097-4

**Published:** 2017-12-06

**Authors:** T. J. Broomhall, T. J. Hayward

**Affiliations:** Department of Materials Science and Engineering, University of Sheffield, Sheffield, UK

## Abstract

Finite temperature micromagnetic simulations were used to investigate the magnetisation structure, propagation dynamics and stochastic pinning of domain walls in rare earth-doped Ni_80_Fe_20_ nanowires. We first show how the increase of the Gilbert damping, caused by the inclusion rare-earth dopants such as holmium, acts to suppress Walker breakdown phenomena. This allows domain walls to maintain consistent magnetisation structures during propagation. We then employ finite temperature simulations to probe how this affects the stochastic pinning of domain walls at notch-shaped artificial defect sites. Our results indicate that the addition of even a few percent of holmium allows domain walls to pin with consistent and well-defined magnetisation configurations, thus suppressing dynamically-induced stochastic pinning/depinning phenomena. Together, these results demonstrate a powerful, materials science-based solution to the problems of stochastic domain wall pinning in soft ferromagnetic nanowires.

## Introduction

The past decade has seen substantial research into the development of memory and logic devices where data is encoded using the positions of domain walls in soft ferromagnetic nanowires^1–4^. These devices employ the motion of DWs though the nanowires to transport data, which can be achieved either through the application of external magnetic fields or electric currents^5,6^. However, the application of even modest magnetic fields or current densities typically lead to Walker breakdown (WB) phenomena^7^, which introduce new levels of complexity to DW motion through periodic oscillations in their internal magnetisation structure^7–9^. When combined with thermal perturbations, these changes in structure impart an inherent stochasticity to DW motion^10^ and thus, as well reducing DW velocities, result in stochastic pinning and depinning at both artificial and intrinsic defect sites, due to the pinning of different meta-stable DW configurations^10–12^. The role of stochastic behaviour in limiting the reliability of DW data storage and logic devices means that understanding and controlling these complex dynamical processes is of major importance for future applications and proposed devices.

One clear route to enhancing the reliability, and thus feasibility, of DW devices is to directly supress the WB phenomena that lie at the heart of stochastic behaviour. A number of methods of suppressing WB have been previously proposed including the patterning of controlled nanowire edge profiles^13,14^, applying transverse magnetic fields^15^, inducing perpendicular magnetic anisotropies^16^, or increasing the Gilbert damping parameters, α, of the materials from which the nanowires are formed^17^. For the latter, increased values of α in soft materials such as Ni_80_Fe_20_ can be obtained by doping with a few percent of rare earth (RE) metals such as terbium or holmium^18,19^ which increase the strength of spin-orbit interactions and thus the rate at which energy is dissipated by precessing spins^17,18^. Typically, the magnetic moments of the RE dopants couple antiferromagnetically to those of the Ni and Fe atoms, resulting in ferrimagnetic order within the lattice^20^. Thus, in addition to modifying α, rare earth doping also causes a net reduction of M_S_ relative to that of undoped films. This is expected to further modify DW structure and dynamics through a relative reduction of the nanowires’ dominant shape anisotropy.

In this paper, we use micromagnetic simulations to explore the magnetisation structure, propagation dynamics and stochastic pinning behaviours of DWs in Ni_80_Fe_20_ nanowires doped with 0–10% of Ho. We first investigate how the reduction of M_S_ resulting from the doping modifies the relative stability of transverse DWs (TDW) and vortex DWs (VDW) in nanowires of a variety of geometries. We then go on to study how corresponding increases in the Gilbert damping constant, α, increase the Walker breakdown field, such that the dynamics of DW propagation are stable over an increased range of driving fields. Finally, we use finite temperature micromagnetic simulations of DWs pinning at artificial defect sites to demonstrate how changes in DW propagation dynamics can be used to suppress the stochastic pinning effects. Together, our results demonstrate the feasibility of a materials science-based solution to the problems of stochastic DW behaviour. If experimentally realised, this could be applied to create devices where DW behaviour is intrinsically well-defined.

## Effect of Rare Earth Doping on Ground State Domain Wall Structures

**Figure 1:**
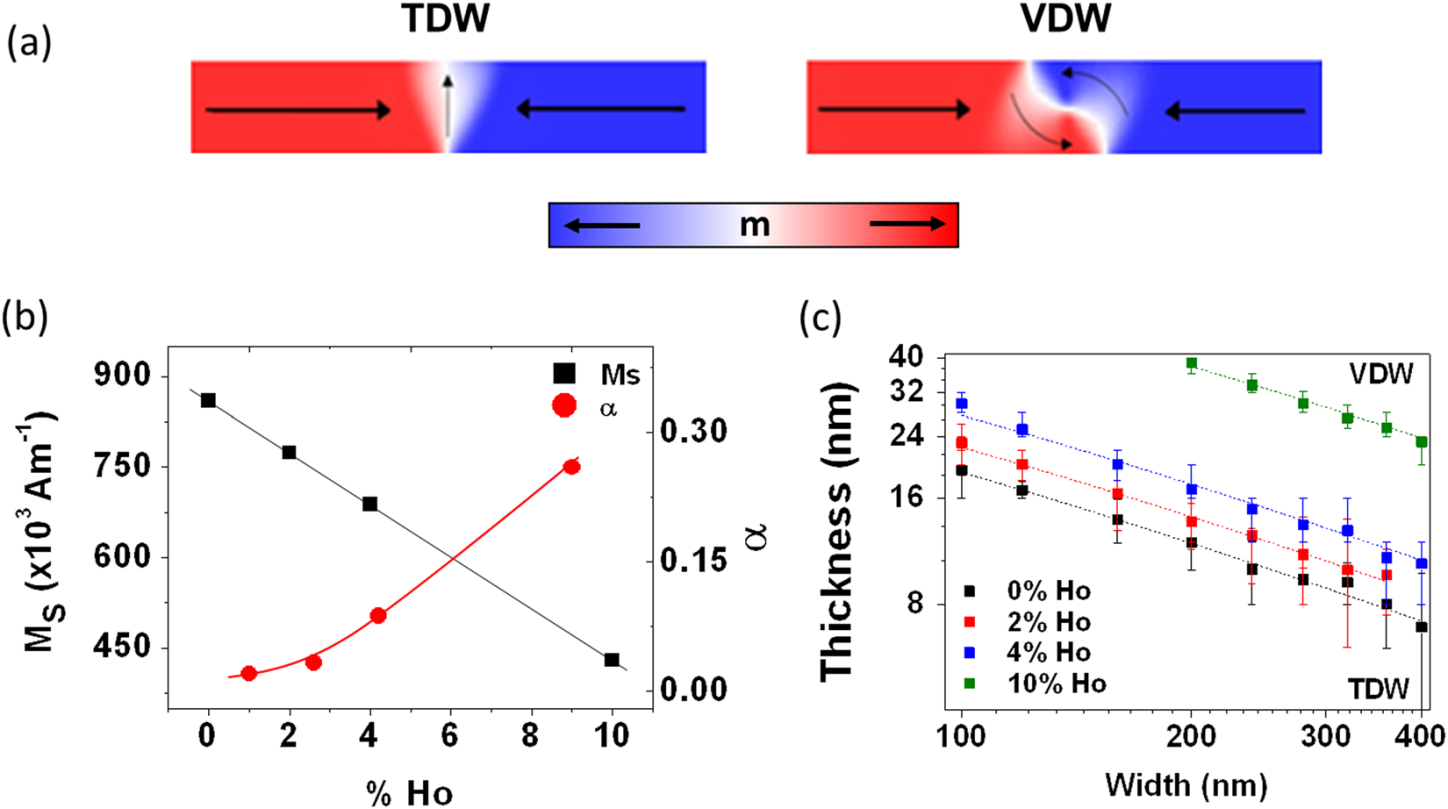
(**a**) Presents example magnetisation configurations for transverse (TDW) and Vortex (VDW) DWs. (**b**) The values for α and M_S_ for each doping value as found in ref.^20^, The solid lines are guides to the eye. (**c**) Phase diagram indicating the stability of TDWs and VDWs for a range of width and thicknesses with different values of holmium doping. The dashed lines are fits using equation 2.

The magnetisation structures of DWs in planar nanowires depend on the balance between exchange and magnetostatic energies. Exchange interactions favour transverse domain wall configurations (TDW), whereas the magnetostatic energy favours vortex domain wall (VDW) configurations (Fig. 1(a)). Several studies have investigated how the relative energies of these DW structures depends on nanowire wire geometry and have shown that VDWs are favoured in thicker and wider nanowires, where magnetostatic energy dominates^21^. TDWs are favoured in thinner and narrower wires where exchange energy plays a more significant role^22^. In Ho doped nanowires, this geometric dependence is further complicated by the reduction of saturation magnetisation resulting from the antiferromagnetic coupling of rare earth atoms with the dominant ferromagnetic phase. This modifies the strength of magnetostatic interactions, and thus the relative stability of VDWs and TDWs compared to un-doped nanowires with the same geometries^21,23^. This would be expected to affect stochastic pinning/depinning by changing both the basic magnetisation dynamics of Walker breakdown^8^, and the relative stabilities of the different DW structures that may be pinned at a given defect site.

To investigate the effects of Ho doping on ground state DW structure, the energies of TDWs and VDWs were simulated in Ho-doped nanowires with widths, w, in the range 100–400 nm and thicknesses, t, in the range 4–40 nm. Values of α and M_S_ for Ho-doped Ni_80_Fe_20_, have previously been reported by Moore *et al*.^20^ (Fig. 1(b)), and were used to model nanowires doped with 0, 2, 5 and 10% Ho. Once the energies of TDWs and VDWs were found for each nanowire geometry the data was interpolated to determine a geometric phase boundary separating the two ground state DW configurations at each dopant level.

Plots of the phase boundary separating TDW and VDW ground states are shown in Fig. 1(c). These obeyed the form originally suggested by McMichael and Donahue^21^, and reproduced experimentally by Kläui *et al*.^23^, with VDWs being favoured in nanowires with large widths and thicknesses, and TDWs being favoured in narrower or thinner nanowires. The mathematical form of the phase boundary, Equation (1), is derived in ref.^21^:

1 $$t=\frac{16\,\pi \,\mathrm{ln}\,(\frac{{r}_{max}}{{r}_{min}})\frac{A}{{\mu }_{0}{M}_{S}^{2}}}{w}$$

where *A* is the material’s exchange stiffness, *M*_*s*_ is the material’s saturation magnetisation, *r*_*max*_ is the outer radius of the VDW and *r*_*min*_ is the radius of the DW’s vortex core (of the order of an exchange length). To evaluate the data presented here we fit a semi-analytical form, Equation (2):

2 $$t=\frac{16\,\pi \,\mathrm{ln}\,(\frac{kw}{{\delta }_{ex}})\frac{A}{{\mu }_{0}{M}_{S}^{2}}}{w}$$

Here, we have approximated $\frac{{r}_{max}}{{r}_{min}}=k\,\frac{w}{{\delta }_{ex}}$, where ${{\delta }}_{{\rm{ex}}}=\sqrt{\frac{2{\rm{A}}}{{{\rm{\mu }}}_{0}{{\rm{M}}}_{0}^{2}}}$ is the exchange length and k is a scaling parameter. Due to the slow variation of the logarithmic term with w, an approximately linear phase boundary is expected on a log-plot, as is observed for the data in Fig. 1(c).

For an undoped nanowire the phase boundary is close to linear and fits the analytic model, Equation (2), well, with the transition point between TDW and VDW located between *t* = *18* ± *2 nm* for w = 100 nm, which is similar to previously found values^23,24^. The scaling parameter, *k*, was found to be 0.79 ± 0.02 suggesting that relating *r*_*max*_ and *r*_*min*_ to the nanowire width and exchange length are reasonable approximations^21^. When the Ho % level was increased, the same basic trend was observed but with TDWs being stable over a greater range of thicknesses and widths. The value of *k* was found to be 0.78 ± 0.06, 0.81 ± 0.04, and 0.73 ± 0.01 for 2, 4 and 10% Ho doped permalloy respectively. These values of *k* are similar to those found for 0% Ho indicating a similar relationship between *r*_*max*_ and *r*_*min*_ across all doping levels.

The increasing stability of TDWs found with increasing Ho % reflects the reduction of M_S,_ and thus magnetostatic energy, in the doped nanowires, which decreases the energy of TDWs relative to VDWs. Notably, for undoped nanowires, both TDWs and VDWs are stable over a wide range of experimentally typical nanowire geometries (100–500 nm wide, 5–40 nm thick), but when even small percentages of Ho are present it became increasingly harder to stabilise VDWs. For example, in the case of 10% Ho doping, only extremely wide and thick (>300 m wide and >40 nm thick nanowires) would be expected exhibit a VDW ground state.

We note that our modelling assumes that Ho doping modifies only the values of α and M_S_. However, Benatmane *et al*.^25^ have previously shown that the presence of RE dopant ions will also enhance the material’s magnetocrystalline anisotropy, which could potentially affect the DW phase diagram^26^. However, for the Ho doping levels similar to those we study here (<10%) the enhancement of the magnetocrystalline anisotropy constant, K_1_, is weak and no increase in anisotropy is seen between 4% Ho and 10% H^25^, hence our decision to neglect it in our modelling. To validate this approach, we repeat our calculations for a 4% Ho-doped nanowire, for which a reported value of K_1_ = 619 J.m^−3^ is calculated from ref.^25^. We re-calculated the energy of TDW and VDW configurations with a uniaxial magnetocrystalline easy axis orientated along both the nanowires long and short axes, and found the differences in the position of the phase boundary to be lower than the cell size of the simulations. This suggested that the effects of a dopant induced magnetocrystalline anisotropy of this magnitude were negligible when compared to those resulting from changes in M_S_, which dominate the energy contributions to the nanowire’s ground DW states.

## Effect of Doping on Domain Wall Dynamics

**Figure 2:**
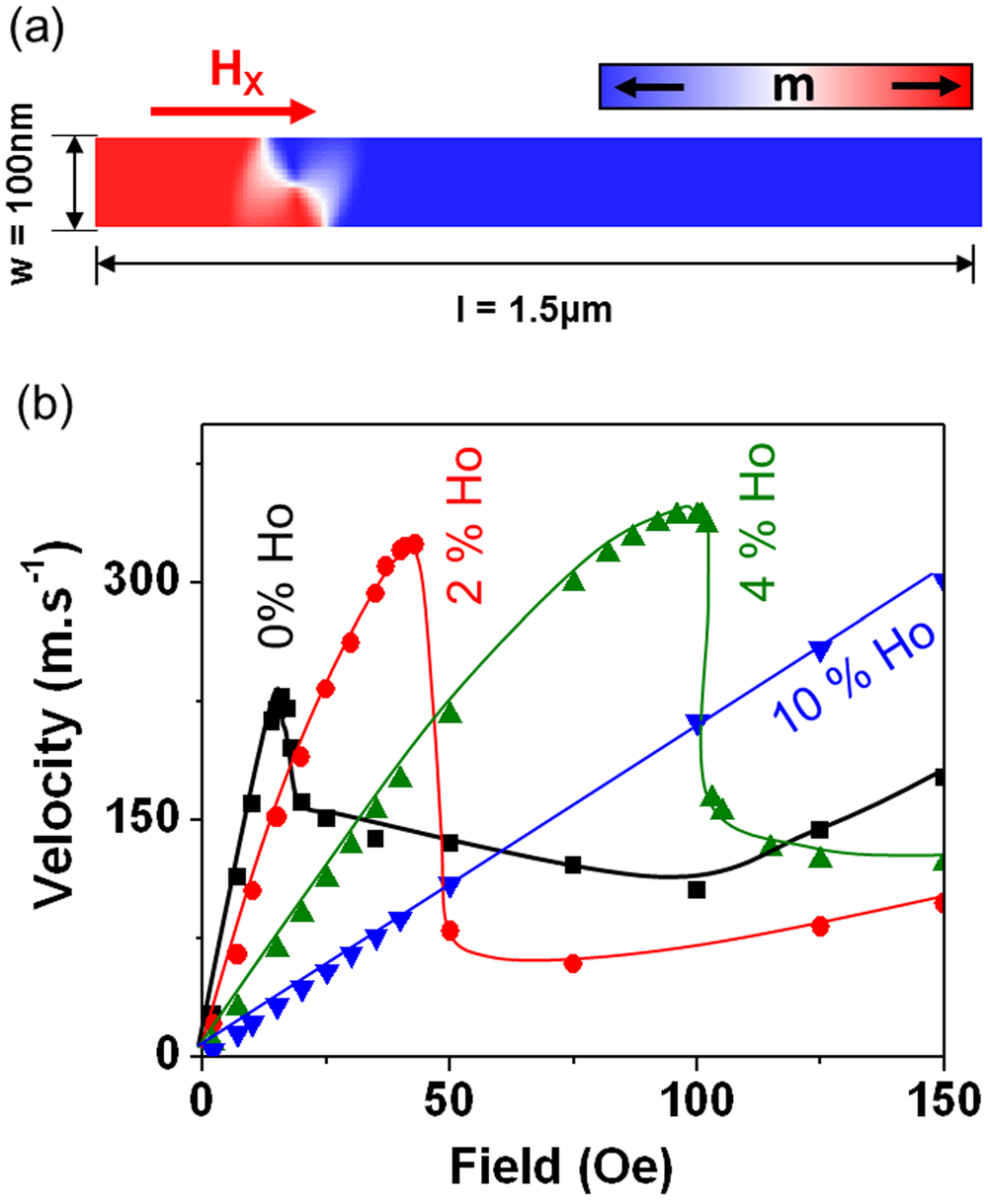
(**a**) Schematic showing the geometry of the nanowires used for simulation of DW propagation, (**b**) Simulated values of DW velocity as a function of applied field for nanowires doped with 0% (squares), 2% (circles), 4% (up triangles) and 10% (down triangles) holmium. The sharp decrease in velocity indicates the onset of Walker breakdown. The solid lines are guides for the eye.

Having determined the effects of Ho doping on the magnetisation structure of DWs, we turned our attention to understanding how Ho concentration modifies the dynamics of propagating DWs. We focused in particular on how the resulting increases in α suppressed the onset of WB, such that it occurs at higher fields. All simulations in this section were performed for nanowires with length = 1.5 µm, width = 100 nm, thickness = 20 nm. DWs were initialised into the nanowires with magnetisation configurations equivalent to those previously determined to be the ground state for a nanowire of this geometry for each doping level (Fig. 1(c)). The geometry of the simulations is illustrated in Fig. 2(a). Initial studies into the effects of realistically sized magnetocrystalline anisotropies show that, as in the static simulations described above, these had a negligible effect on the magnetisations dynamics. Therefore, all simulations were performed with K_1_ = 0, with the effects of doping being reflected solely through changes in α and M_s_.

We first considered the 1-dimensional model of DW motion^7,15^. This describes the motion of a DW under a constant applied field and can be applied to calculate the value of the Walker breakdown field and the maximum velocity attainable within the linear regime. The value of H_WB_ is defined in equation 3, as a function of the saturation magnetization, M_S_, and the damping factor, α:

3 $${H}_{WB}=\frac{{M}_{S}\alpha }{2}$$

Thus, increases in both M_S_ and α can increase the value of H_WB_, thereby supressing the onset of Walker breakdown. The DW velocity in the viscous regime is given by:

4 $$\nu =(\frac{\gamma {\rm{\Delta }}}{\alpha })\,H$$

where γ = 2.2 × 10^5^ m.A^−1^ s^−1^ is the gyromagnetic ratio, Δ is the DW width, and H is the applied field. The model predicts that the DW velocity is proportional to the H and ${\rm{\Delta }}$, and inversely α. Combining equations 3 and 4 allows prediction of the maximum velocity in the viscous regime (i.e. that directly preceding Walker breakdown):

5 $${\nu }_{max}\,=\,\gamma {\rm{\Delta }}{M}_{S}$$

This equation shows that, in the 1D model the maximum velocity is not dependent upon the value of damping, but would be proportional to M_S_ and the domain wall width Δ.

To simulate the effect of Ho doping on Walker breakdown, DWs were propagated at applied fields in the range, 2 Oe–400 Oe, and their velocities calculated from plots of M_x_/M_s_ vs time. Modes of DW propagation can be characterised by the DW velocity, which progresses through three clearly defined regimes as the applied field is increased. At low field strengths, the viscous regime of motion occurs, where DWs propagate rigidly and the velocity is linearly proportional to the applied field, as described by equation (4). This viscous regime terminates at the onset of Walker breakdown (WB), the field at which this occurs is defined as H_WB_, which in the 1D model may be calculated using equation (3)_._ During WB, domain walls begin to undergo periodic changes in structure, causing a dramatic decrease in their propagation velocity. This is known as the oscillatory regime of motion, and further increases in applied field in this regime typically cause the DW velocity to plateau. Finally, a point is reached whereby upon further increasing the applied field the DW velocity again begins to increase as the DW enters the turbulent regime of motion. Here, the DW is still undergoing Walker breakdown transformations, however the applied field is sufficient enough to overcome the reduction in velocity these produce, and the transformation cycles become much less predictable^10^.

DW velocities as a function of applied field for each of the simulated nanowires are shown in Fig. 2(b). For an undoped nanowire the linear regime terminated at 16 Oe with the onset of WB (H_WB_), and progressed into the oscillatory regime, which occurred at fields between 16 Oe and 100 Oe. Beyond this field range the DW motion fell within the turbulent regime and the velocity again began to increase. When increasing the Ho doping level to 2% the linear regime occurred at fields up to 43 Oe, and the oscillatory regime occurs between 43 Oe and 125 Oe. For Ho = 4%, the linear regime terminated at an applied field of 101 Oe, while for Ho = 10% the linear regime ended at 275 Oe. For these latter two dopant levels the turbulent regime of motion occurred beyond the simulated field range.

**Figure 3:**
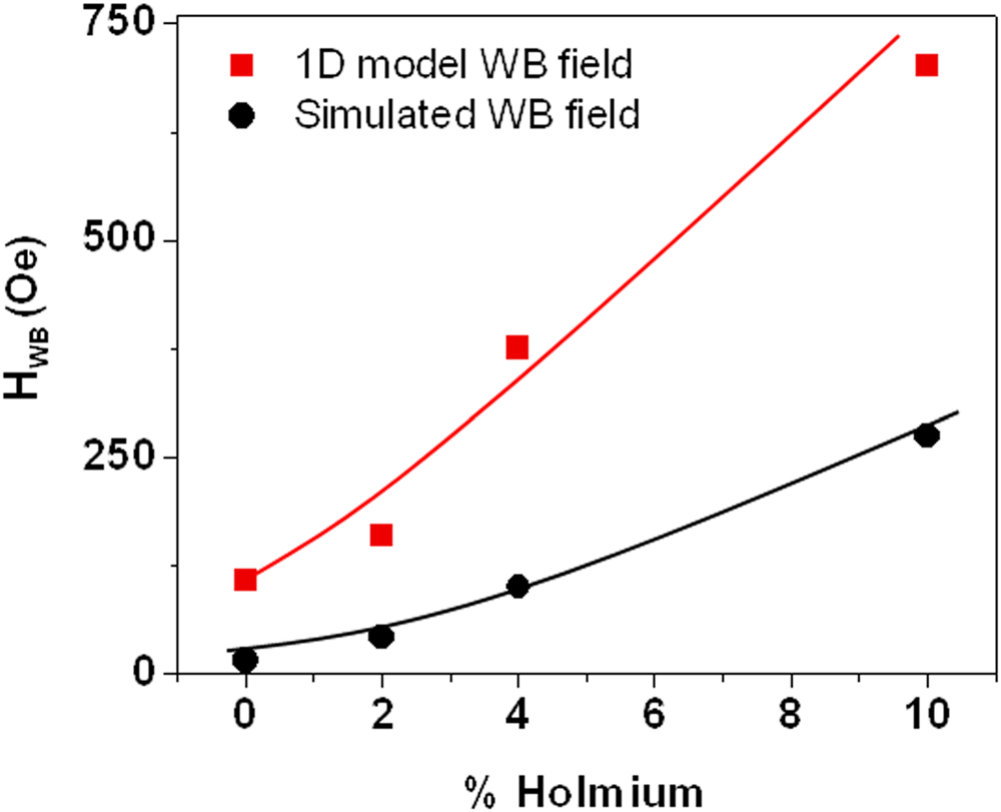
Values of the Walker breakdown field (H_WB_) as a function of % holmium as simulated (circles) for 100 nm wide, 20 nm thick nanowires and as calculated from the 1D model (squares). The solid lines are guides for the eye.

Figure 3 presents the calculated values of H_WB_ from our simulations and compares them with values derived analytically from equation (3). By increasing the Ho-doping level from 0 to 10%, H_WB_ was enhanced by over an order of magnitude. Therefore, the inclusion of small quantities of Ho increases the linear regime of DW motion, resulting in domain walls being propagated with consistent DW configurations across a larger range of applied fields. The velocity of a domain wall at H_WB_ was found to increase by approximately a factor of 2 (from 227 ms^−1^ to 422 ms^−1^) from the undoped nanowire to that with the maximum dopant level. The 1D model of DW motion also suggested that H_WB_ will increase with increasing Ho doping level, as the change in α varies more rapidly than the value of M_S_ decreases, however our simulated values of H_WB_ were consistently lower than those predicted by the model. We note that analytical models of DW motion have previously been found to be produce qualitative agreement but quantitative disagreement with the results of micromagnetic simulations^27,28^.

**Figure 4:**
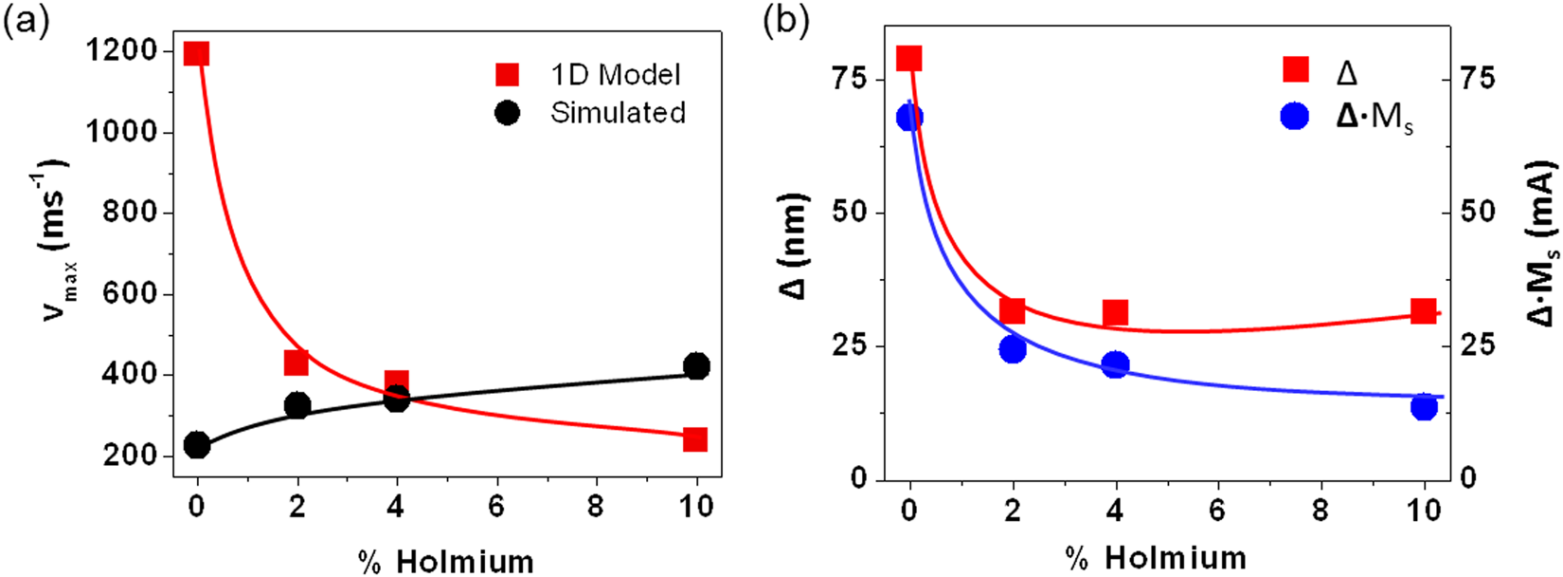
(**a**) Maximum DW velocity prior to Walker breakdown observed in 1D model (red squares) and micromagnetic simulations (black circles) as a function of holmium doping level. (**b**) Plot showing fitted DW width parameter, ∆ (red squares), and value of ∆·M_s_ (blue circles) as a function of holmium doping level. The solid lines are guides for the eye.

Our simulations also indicated that the maximum velocity within the linear regime increased with increasing dopant level (Fig. 4(a)). The 1D model predicts that the maximum velocity depends upon M_s_, but also on the domain wall width parameter ∆. Thus, to compare maximum DW velocities predicted by the 1D model with our simulated data, we measured the domain wall width parameter from the relaxed ground state domain wall configurations simulated by fitting the function $\,{M}_{x}/{M}_{S}=\pm \,\tanh (x/{\rm{\Delta }})$ to the width averaged magnetization profile of the DWs (Fig. 4(b))^24^. It was found that the domain wall width decreased by a factor 2 across the simulated range of doping levels. This is primarily due to the change of ground state from VDW (undoped nanowire) to TDW (doped nanowires), with a VDW having a width approximately twice that of a TDW. In combination with the reduced M_s_ values in the doped nanowires, the decreasing value of Δ with increasing Ho doping levels, means that equation 4 predicts a decrease in the maximum velocity of the DWs, in direct contrast to the simulated results (Fig. 4(a)). Clearly, the 1D model was incapable of capturing these subtle features of DW motion in systems with complex, compositionally varying DW spin structures. Thus, we conclude that while the 1D model offers a good phenomenological description of DW motion in rare earth-doped nanowires, for example predicting an increase in H_WB_ and a decreased mobility in the viscous regime with dopant level, it is not capable of quantitatively predicting DW velocities^21^, and in some cases will produce trends inconsistent with experimental data (e.g. variation of max DW velocity) due to its over-simplification of DW structure and dynamics.

A further point of interest from our simulations is that for a given DW propagation field there is an optimised dopant level that will provide the maximum DW velocity (Fig. 2(b)). For example, for fields <19 Oe the undoped nanowires showed the greatest velocities due to the reduced mobilities of the doped nanowires. For fields in the range 19–46 Oe the 2% Ho doped nanowires had the highest velocities, due to the undoped nanowires having fallen into the oscillatory regime of motion. Similarly, for fields in the range 46–102 Oe the 4% Ho doped nanowires exhibited the highest velocities. Finally, the 10% doped nanowires were optimised for propagation fields> 102 Oe. This is an interesting and somewhat counter-intuitive result, as it is generally presumed that higher values of α reduce the velocities of DWs, whereas our results show this is not necessarily the case.

**Figure 5:**
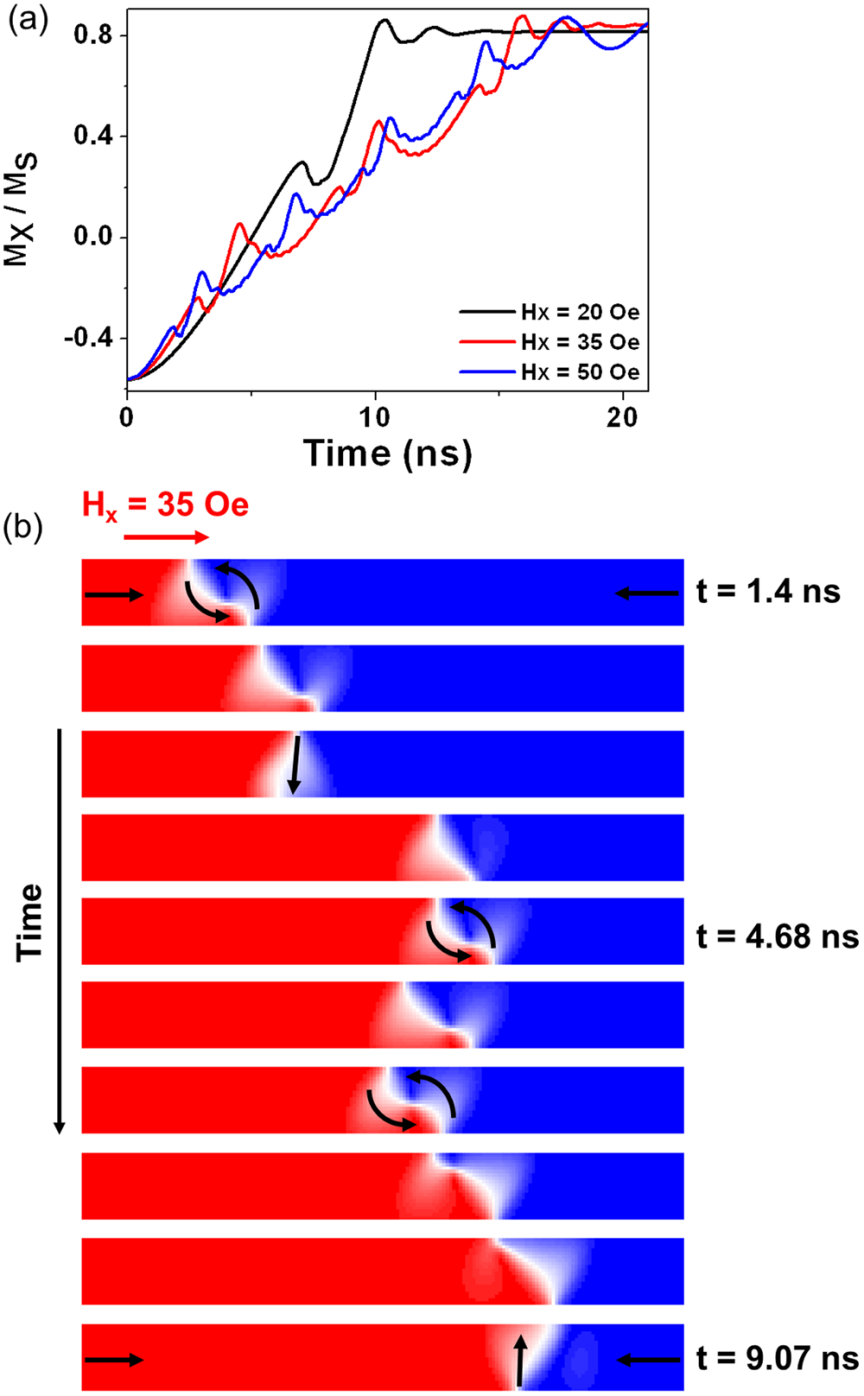
(**a**) M_x_ vs time plot for a 0% holmium nanowire under applied fields of 20, 35 and 50 Oe. (**b**) shows the WB profile for 35 Oe, illustrating a VDW–TDW–VDW–TDW* transformation sequence.

**Figure 6:**
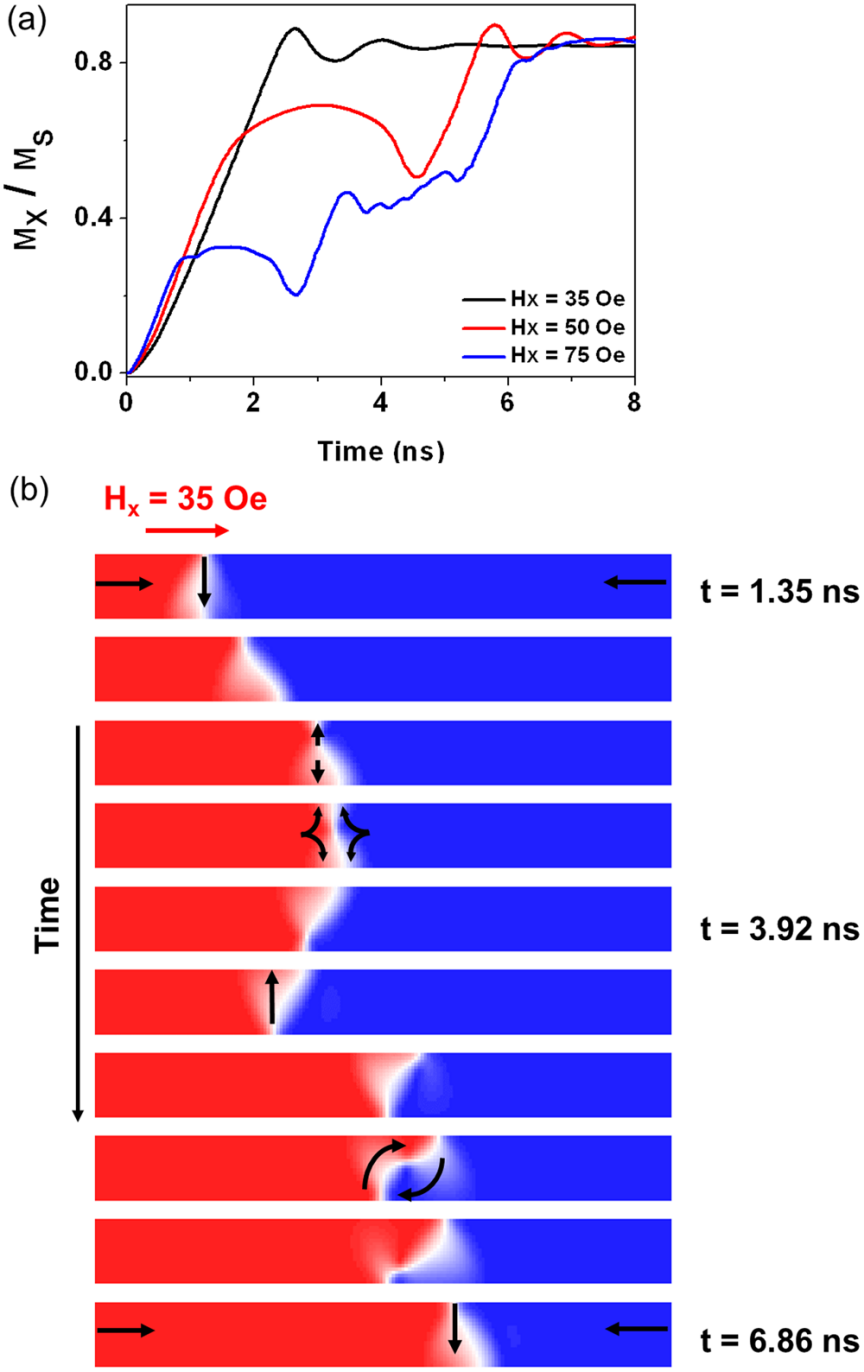
(**a**) M_x_ vs time plot for a 4% holmium nanowire, under applied fields of 35 Oe, 50 Oe and 75 Oe. (**b**) shows the WB profile at 75 Oe, illustrating–TDW–aVDW–TDW* - VDW transformation sequence, in clear contrast to the dynamics observed for the undoped nanowire.

In addition to the changes in DW velocities, we also observed that increased levels of doping fundamentally modified the magnetisation dynamics of DWs propagating above WB. This can be seen in the M_x_/M_s_ vs time plots in Figs 5(a) and 6(a), which illustrate the propagation behaviours of DWs in a undoped, and a 4% Ho-doped nanowire respectively. For an undoped system the M_x_/M_s_ vs time plots show a number of distinct crests and troughs indicating a change in DW configuration (Fig. 5(a)). With increasing applied fields these periodic oscillations increase in frequency, with the shape of these crests and troughs remain constant. This indicates the same oscillatory behaviour in DW configuration at all fields post H_WB._ Upon increasing Ho dopant levels, similar features are seen in M_x_/M_s_ plots; periodic crests and troughs describing the Walker breakdown profile of these doped nanowires. However, the periodic features exhibit different shapes (Figs 5(a) and 6(a)), indicating different transformation sequences of the DWs.

The differences in DW response during propagation is further illustrated in Figs 5(b) and 6(b), which show the micromagnetic configurations of DWs propagating above WB in an undoped and a 4% Ho-doped nanowire. In the undoped nanowire, the WB dynamics can be described as a VDW–TDW–VDW–TDW* transformation sequence (with the * representing a DW of opposite chirality). However, at increased values of Ho-doping the WB mode changes to include anti-vortex DWs (aVDWs) in place of VDWs. For example, at 4% Ho-doping (Fig. 6(b) the aVDWs appear metastable with the VDWs and both are seen within the WB profile (TDW–aVDW–TDW* - VDW). The transformation sequence changes again at 10% Ho-doping, where no VDWs were seen during propagation, having been replaced entirely by aVDWs (TDW–aVDW –TDW* - aVDW). This change in WB dynamics, causes the differences in shape of the M_x_/M_s_ vs time traces, and can be attributed to the reduction in the stability of VDWs at increased doping levels. We note that similar changes in the nature of WB dynamics have been observed as a function of nanowire geometry in previous studies^8^, and can similarly be attributed to variations in the stability of the DW structures observed during the WB transformation sequences.

The results presented in this section indicate that doping NiFe nanowires with rare earth materials pushes the onset of WB towards higher field values, thus allowing consistent DW configurations to be maintained during propagation across a larger range of applied fields, and also substantially modify the form of WB dynamics once H_WB_ is exceeded. In the following section, we shall use finite temperature simulations to show how the former property allows DWs in doped nanowires to show more reliable responses when they interact with defect sites.

## Effects of Doping on Stochastic Domain Wall Pinning

Having established the that doping Ni_80_Fe_20_ nanowires with Ho pushes the onset of Walker Breakdown towards higher fields, we investigated how this affected the stochastic pinning of DWs at artificial defect sites. To do this, we follow the approached used in ref.^10^ where room temperature micromagnetic simulations were used to show how experimentally observed stochastic pinning behaviours can be explained by the interaction of thermal perturbations with post-WB DW dynamics.

Simulations were performed in nanowires with the same dimensions as in the previous section but with artificial defects in the form of triangular notches located in the nanowires’ upper edges at distances of 1.25 µm from their left-hand edge. Ground state DWs were stabilised 300 nm from the left-hand side, and propagated towards the notch under an applied field of 35 Oe, which is a typical DW propagation field in nominally defect free nanowires^29^. As in the work described in ref.^10^, room temperature thermal perturbations were modelled using the method of Brown. For each value of % Ho doping, a deterministic T = 0 K simulation was performed to establish the baseline behaviour of the system, followed by 20 simulations at T = 300 K to investigate the stochasticity of the DWs pinning. Each of the T = 300 K simulations were initialised with a unique seed to randomize the thermal fluctuations that occurred at each time step throughout the simulation. Depinning field distributions (DFD) were derived by measuring the depinning field for each pinned state observed at T = 0 K, and assigning each of these depinning fields a Gaussian distribution with standard deviation (σ) equal to 1 Oe^10^. The simulations of depinning were performed quasi-statically (α = 0.5) to reduce computation time.

**Figure 7:**
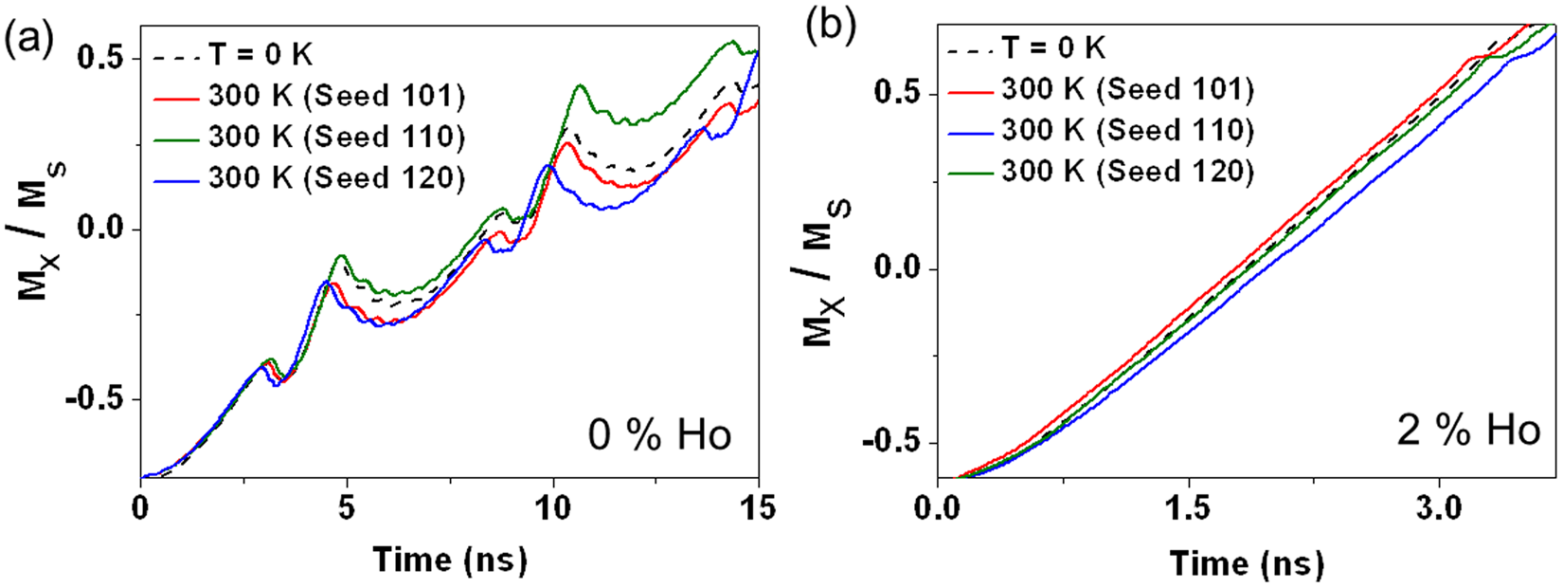
M_x_/M_s_ vs time plots for (**a**) 0% holmium and (**b**) 2% holmium nanowires under a propagation field of 35 Oe. Each plot shows both a deterministic T = 0 K run (dashed lines), and 3 runs at T = 300 K with different random thermal seeds (full lines).

Figure 7 illustrates how thermal perturbations can modify DW propagation at H = 35 Oe in both an undoped and a 2% Ho doped nanowire. H = 35 Oe is a typical propagation field for devices and is above H_WB_ in an undoped nanowire, and below H_WB_ in the 2% doped nanowire. In the instances where a DW was propagated through an undoped nanowire, the thermal perturbations primarily affected the transition between DW configurations in the Walker breakdown sequence. This gave rise to a greater variety of time averaged velocities in the undoped nanowire. These differences are highlighted in Fig. 7(a) where the M_X_ vs time plots show WB transitions beginning at different times, and requiring different lengths of time to stabilise into the next configuration within the sequence. In contrast, DWs propagated through a 2% Ho doped nanowire (Fig. 7(b)) showed less variance in the time averaged DW velocity, as they propagated in the viscous regime where there are no transitions between DW states. We note that these results are consistent with those in ref.^10^ where it was found that thermal perturbations only have significant effects on DWs propagating above the H_WB_.

**Figure 8:**
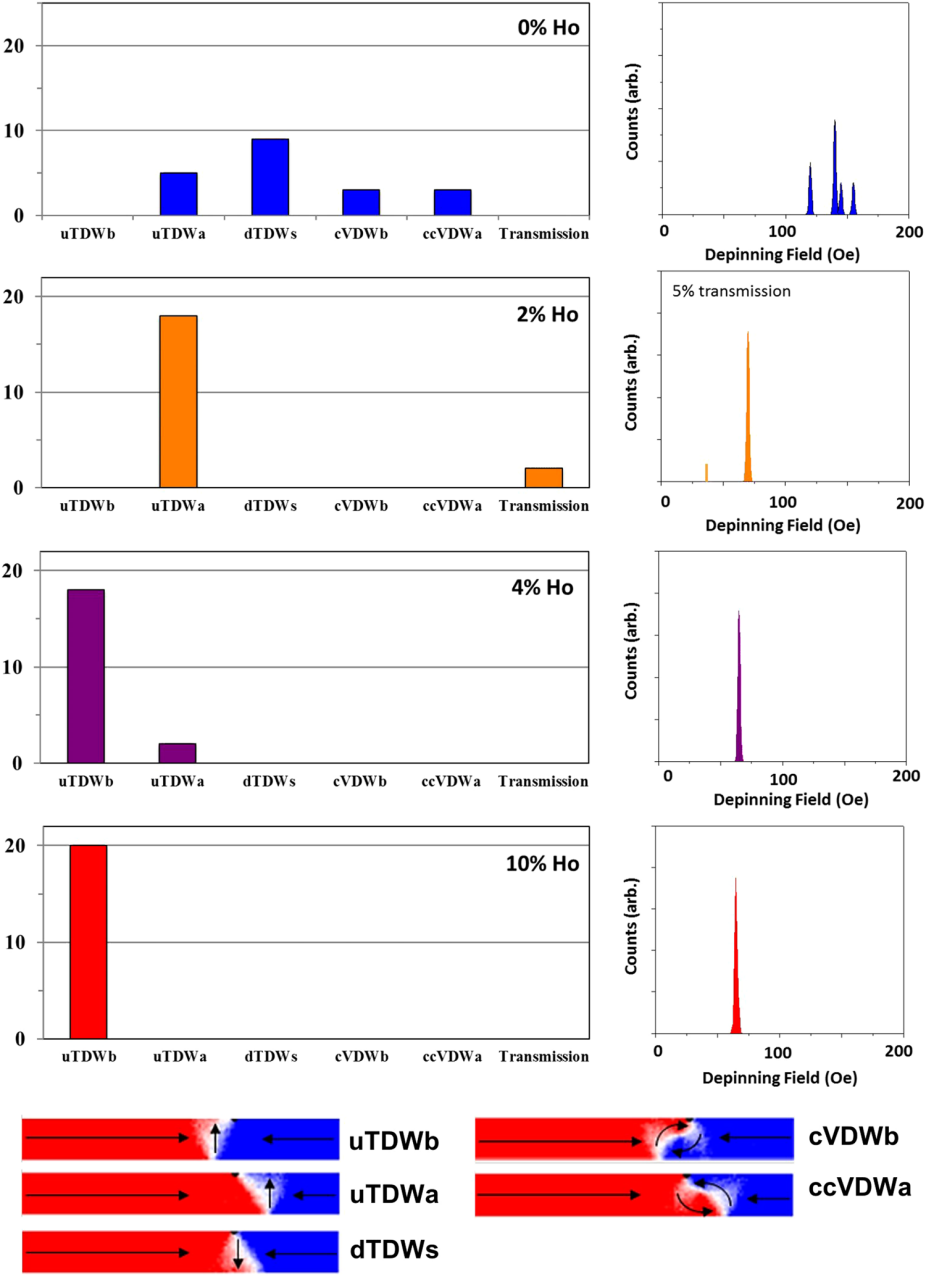
Distributions of pinned states found for nanowires containing an 18 nm width/depth notch and holmium concentrations of 0, 2, 4 and 10%. DWs were propagated to the notch at H = 35 Oe. The figures on the right illustrate depinning field distributions derived from quasi-static simulations of each pinned state’s depinning fields. Example magnetisation configurations for each of the pinned states are shown at the bottom of the figure.

Figure 8 presents the distribution of pinned DW states and derived DFDs for DWs propagated towards an 18 nm deep notch in nanowires doped with 0%, 2%, 4%, 10% Ho. In the undoped nanowire four distinct pinned DW states were identified; up TDWs (uTDW) pinned before the notch, down TDWs (dTDWs) located symmetrically within the notch, cVDWs located before and aVDWs pinned after the notch. The variety of states arising are due to randomising influence of thermal effects on the DW transformation sequence^10^ described above. The four pinned states gives rise to an equal number of distinct depinning fields, causing a wide depinning field distribution, similar to those that have been found experimentally for notch-shaped defect sites^30^.

For the 2% Ho doped nanowires we observed two distinct outcomes; an uTDW pinned after the notch, and 2 instances where the DW passed through the notch without pinning. When Ho doping was raised to 4% two pinned states were observed: a majority of cases where an uTDW pinned before the notch and a minority where an uTDW pinned on the notches other side. As uTDW was the initial DW configuration in these simulations this indicated that no changes in DW configuration occurred, which is consistent with WB being suppressed at this propagation field. However, the presence of two distinct pinned states again showed evidence of sub-WB stochasticity. In contrast to this when Ho = 10%, a single pinned configuration was observed (uTDWb), indicating a complete suppression of stochasticity.

The derived depinning field distributions found for values of Ho = 2%, 4%, 10% clearly show less complexity and stochasticity than for an undoped nanowire, indicating that Ho doping has substantially reduced the stochasticity of DW pinning. However, some stochasticity remained in nanowires with 2% and 4% Ho, in particular a possibility of the DW passing through the notch and either transmitting to the end of the nanowire (2% Ho); or becoming pinned on the far side of the notch (4% Ho). No WB transformations of DW structure occur in these simulations, and therefore the stochastic effects must have a different origin. We therefore considered two different possibilities for this: (1) small, sub-WB precession within the structure of the propagating DWs and (2) “dynamic” thermal activation of the rigidly propagating DWs over the defect site.

To investigate whether the resulting stochasticity occurred due to sub-WB precession of the DWs structure, T = 0 K simulations were performed where DWs were initialised at different distances from the notch and then propagated towards it at an applied field of H = 35 Oe. This was intended to allow the DWs to interact with the defect site at different points in any precessional cycles that were occurring. For each dopant level, 19 simulations were performed with the DWs starting between 640 nm–1172 nm from the notch, with consecutive simulations moving the initial position by 28 nm, a distance equal to the width of the notch. The total range of initial positions was equivalent to the distance along the nanowire in which a complete WB sequence occurs at an applied field of H = 35 Oe in an undoped nanowire.

For an undoped nanowire, where the DWs propagated above WB, a range of pinned states were found; uTDWs pinned at either side of the notch, dTDWs pinned within the notch, and VDWs positioned either side of the notch. Two instances of transmission of a DW occurred, unlike those found in room temperature simulations, but otherwise these pinned states are similar to those seen in the T = 300 K simulation presented in Fig. 8, verifying that the stochastic nature of pinning seen was primarily caused by the combination of WB phenomena with the randomising influence of thermal excitations. However, for the nanowires with doping levels of 2%, 4%, 10% Ho only one pinned state (uTDWb) was found for all initial DW positions. As the initial DW state pinned in all cases where WB did not occur, these results showed that the stochastic pinning observed for the 2% and 4% Ho doped nanowires did not arise from sub-WB precession of the DW. Therefore the resulting stochastic behaviour was likely to be entirely due to “dynamic” thermal activation of the DW through the energy barrier presented by the notch.

To further probe the effects of thermal perturbations on DW pinning below WB, we performed a further set of T = 300 K simulations, with notches with an increased depth of 36 nm. Increasing the depth of the notch was expected to increase the strength of its pinning potential^31^, and thus to help suppress thermal activation effects at the notch. We anticipated that this would manifest as a complete suppression of stochastic behaviour for cases where the propagation field of 35 Oe was below H_WB_ due to presence of Ho doping.

**Figure 9:**
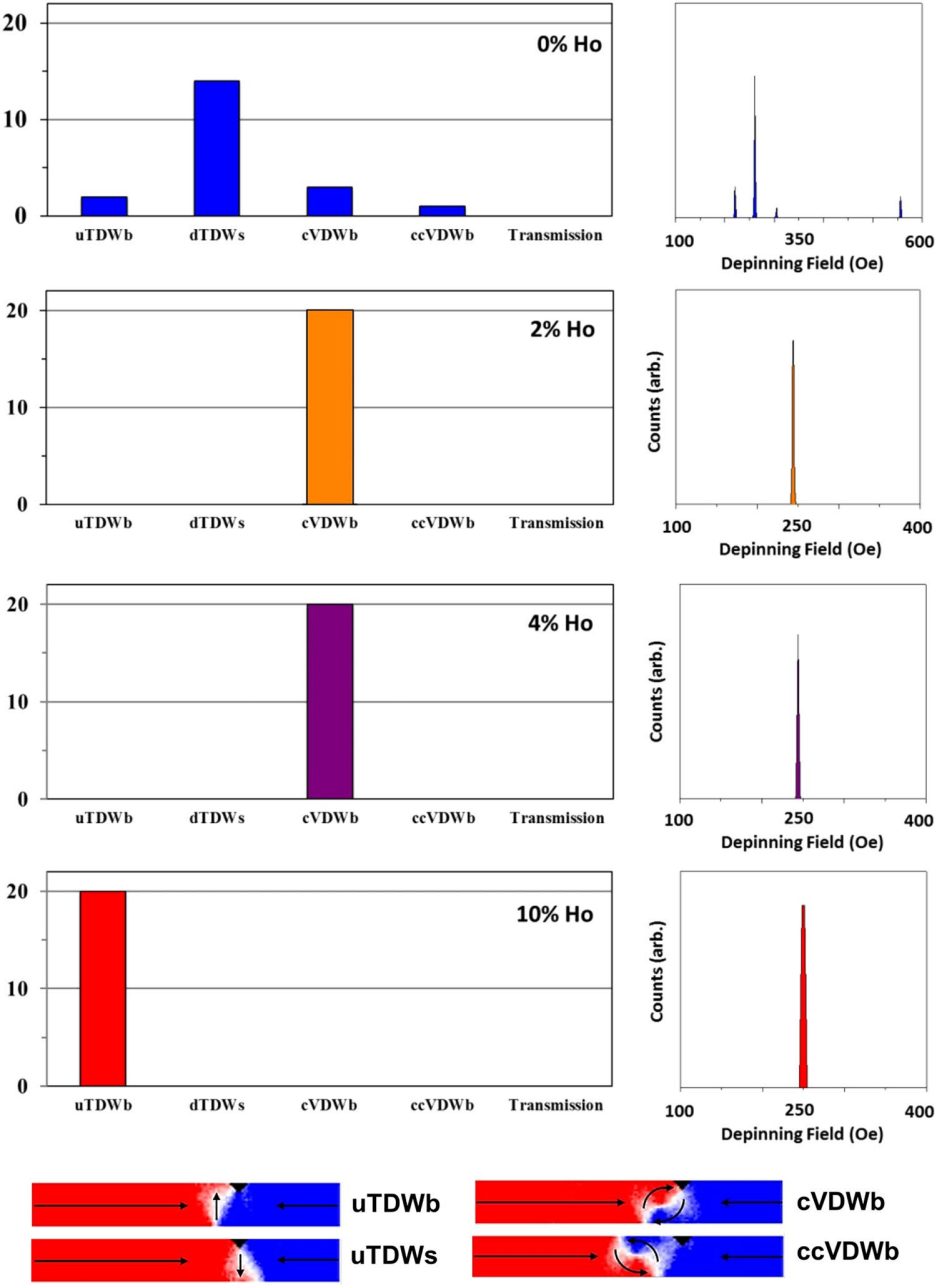
Distributions of pinned states found for nanowires containing a 36 nm width/depth notch and holmium concentrations of 0, 2, 4 and 10%. DWs were propagated to the notch at H = 35 Oe. The figures on the right illustrate depinning field distributions derived from quasi-static simulations of each pinned state’s depinning fields. Example magnetisation configurations for each of the pinned states are shown at the bottom of the figure.

Figure 9 shows distributions of pinned states, and derived DFDs, as a function of the Ho-doping level for a 36 nm notch. For undoped nanowires, we found a reduced level of stochasticity compared to the smaller notch, resulting in one DW configuration (dTDW), becoming the dominant pinned state. Small populations of other pinned states were also present, which again arose from the interplay of thermal perturbations with WB phenomena. This stochasticity manifested as a relatively wide DFD. For the doped nanowires, where WB was supressed at the propagation field, the increased notch size gave rise to deterministic pinning, whereby only one pinned DW state was formed. We believe that this occurred because the strength of pinning potential generated by this larger notch geometry was large enough that thermal perturbations could no longer overcome its energy barrier. We note that for 2% and 4% Ho doped nanowires the DWs pinned as cVDW located before the notch, rather than as the TDWs that were the expected ground state. This change in DW configuration was likely caused by the local shape anisotropy at the notch^32^, which changed the ground state of the pinned DWs, compared to unpinned ones. This change in DW state was not seen for 10% Ho nanowires, which we believe was due to the large difference between the energies of TDWs and VDWs at this doping level (Fig. 1(c)).

## Conclusions

In this paper, we have used room temperature micromagnetic simulations to show that doping Ni_80_Fe_20_ nanowires with small amounts (<10%) of rare earth elements such as Ho can suppress the stochastic pinning and depinning of DWs at defect sites. Such doping causes increases in the damping factor, α, which pushes the onset of Walker breakdown phenomena beyond typical propagation fields, thus preventing the complex DW dynamics that lie at the heart of stochastic DW pinning. We have also shown that simpler stochastic effects, which are caused by “dynamic” thermal activation of DWs over the potential barriers presented by a defect site, rather than Walker breakdown phenomena, can be supressed by tuning of a defect’s geometry.

Additionally, we have demonstrated that although Ho doping reduces the mobility of DWs in the viscous regime, as has been previously shown experimentally^20^, Ho doping can also increase the maximum velocity that DWs may be propagated at while maintaining rigid magnetisation structure. We find the 1D Walker model^7^ phenomenologically describes the increase in Walker breakdown field that occurs with increased doping, however the model fails to describe the increase in maximum velocity that is found within our simulations. Furthermore, our results indicate that for a given window of applied fields, maximum velocities will be obtained for an optimal level of doping that balances decreases in the mobility of DWs against increases in the Walker breakdown field. Finally, we have shown that doping with Ho also has a significant effect on the relative stability of transverse and vortex domain walls. With an increase in Ho %, and consequent decrease in M_S_, TDWs are found to be stable over a range of geometries that would nominally favour VDWs in un-doped Ni_80_Fe_20_.

We note that the materials parameters used in our modelling are derived from non-spatially resolved measurements of thin films^20^. Thus, our simulations do not take into account any inhomogeneity in the distribution of rare-earth atoms that could, for example, lead to the creation of additional pinning sites for DWs. Experimental measurements will be required to examine if such inhomogeneities exist, and how seriously these affect DW behaviour. None-the-less, our work indicates the feasibility of a powerful, materials science-based solution to the problems of stochastic domain wall pinning and paves the way for a new generation of DW devices where material properties are carefully controlled to simultaneously optimise speed and reliability.

## Methods

T = 0 K micromagnetic simulations to investigate domain wall energies, and domain wall velocity within nanowires were performed using the object orientated micromagnetic framework (OOMMF) package^33^. All simulations were discretized into 4 *nm* × 4 *nm* × 4 *nm* meshes, with the 3*D* mesh used to avoid spatial averaging over the complex 3*D* nature of vortex domain walls. An additional magnetic field was calculated and applied to counter-act the effects of the poles at the end of the nanowires, thereby allowing a pseudo-infinite nanowire to be simulated.

We used the standard value of exchange stiffness (A_ex_) = 13×10^–12^ J/m for Ni_80_Fe_20_ in all simulations. Values for α and M_S_ as a function of Ho % were extracted from ref.^20^, and are presented here in Fig. 1(b).

To investigate the ground state domain wall structure, both vortex and transverse DWs were relaxed at 0 K in nanowires with geometries that favoured either VDW or TDW configuration. These idealized DWs states were then relaxed in nanowires of varying geometries, with widths ranging from 100 nm–400 nm and thicknesses of 4 nm–40 nm and their energies calculated. The point at which the energies of the two DW states became equal was used to identify the point at which they were metastable, and therefore the position of the geometric phase boundary separating the two ground states.

To probe the dynamics of propagating DWs, ground state DW configurations were relaxed at a distance of 300 nm from the left-hand side of a nanowire of geometry width = 100 nm, thickness = 20 nm and length 1.5 µm. Static magnetic fields in the range H_x_ = 2–400 Oe were applied along the length of the nanowire. DW velocities were calculated from the rate of change in the M_x_ component of magnetisation. In cases where DW propagation was below H_WB,_ the velocity was taken as maximum gradient on a plot of M_x_/M_S_ vs t, while in cases where the propagation field was above H_WB_ the velocity was averaged over a full WB cycle.

Stochastic DW pinning at T = 300 K was simulated using the MUMAX^3^ software package^34^ using the methods previously demonstrated in^6^. The nanowire geometry investigated was the same as that for the T = 0 K dynamic simulations, but with triangular notches with depths of 18 nm and 36 nm located in the nanowires upper edge at a distance of 1.25 µm from the left-hand end. The nanowire was discretised into a 2.5 nm × 2.5 nm × 2.5 nm mesh. Ground state DWs were located 300 nm from the left-hand side before being propagated with an applied field of H_x_ = 35 Oe. The magnetostatic fields produced by the nanowire’s ends were subtracted using the inbuilt functionality of the MUMAX^3^ software.

It should be noted that micromagnetic simulations using Brown’s approach to finite temperatures applies a high frequency cut-off to the spin-wave spectrum due to the discretization imposed by the mesh size^35,36^. This can have a strong effect on the simulation of critical temperatures such as the Curie point, but we believe it is less likely to influence room temperature micromagnetic simulations such as those presented here. A more thorough discussion of these limitations can be found in ref.^10^.
